# Autoimmune Thyroiditis Shifting from Hashimoto’s Thyroiditis to Graves’ Disease

**DOI:** 10.3390/medicina59040757

**Published:** 2023-04-13

**Authors:** Narantsatsral Daramjav, Junko Takagi, Hideyuki Iwayama, Kaori Uchino, Daisuke Inukai, Kazuo Otake, Tetsuya Ogawa, Akiyoshi Takami

**Affiliations:** 1 Department of Internal Medicine, Division of Endocrinology and Metabolism, Aichi Medical University School of Medicine, Nagakute 480-1195, Aichi, Japan; 2 Department of Pediatrics, Aichi Medical University School of Medicine, Nagakute 480-1195, Aichi, Japan; 3 Department of Internal Medicine, Division of Hematology, Aichi Medical University School of Medicine, Nagakute 480-1195, Aichi, Japan; 4 Department of Otorhinolaryngology Head and Neck Surgery, Aichi Medical University School of Medicine, Nagakute 480-1195, Aichi, Japan

**Keywords:** Graves’ disease, Hashimoto thyroiditis, autoimmune disease, thyroid antibodies

## Abstract

In 15–20% of cases, Graves’ disease (GD) shifts to Hashimoto’s thyroiditis (HT), while the shift from HT to GD is rare. We present a case of a patient in whom HT shifted to GD, along with a literature review. A 50-year-old woman with myxedema was diagnosed with Hashimoto’s disease due to hypothyroidism and the presence of antibodies against thyroid peroxidase (TPOAb) and thyroglobulin (TgAb); she also had thyroid stimulating antibodies (TSAb) without any signs of GD. Although thyroid hormone replacement therapy improved her thyroid function, 2 months later, hyperthyroidism appeared and did not improve after discontinuation of the replacement therapy. The patient was diagnosed with GD, which improved with antithyroid agent administration. To date, only 50 cases regarding conversion from HT to GD have been reported. The median age is 44 years (range, 23–82 years), and the median time of conversion is 7 years (range, 0.1–27 years). The male-to-female ratio of HT conversion to GD is 1:9, closer to that of regular GD (1:10) than that of general HT (1:18). All patients received thyroid hormone replacement therapy for hypothyroidism due to HT. Continuous evaluation of TSAb levels is recommended in HT, particularly in cases of TSAb-positive and those under replacement, since it may help predict conversion to GD. Evaluating the clinical characteristics of patients with HT preceding GD is crucial to ensure appropriate treatment and reduce the risk of adverse events.

## 1. Introduction

Hashimoto’s thyroiditis (HT) and Grave’s disease (GD) are thyroid-specific autoimmune diseases that exhibit conflicting thyroid hormone dynamics. Approximately 15–20% of patients with GD progress to HT, especially after initiation of antithyroid drug therapy. Hyperthyroidism in GD is caused by stimulatory anti-thyroid stimulating hormone (TSH) receptor antibodies; however, 50–70% of patients with GD have anti-thyroid peroxidase antibodies (TPOAb) and/or antithyroglobulin antibodies (TgAb), characteristic of HT [1,2].

TPOAb and TgAb are clinical markers that lead HT pathology to lymphocytic infiltration into the thyroid tissue and hypothyroidism. Additionally, approximately 10% of HT patients are thyroid stimulating antibodies (TSAb)-positive [3], although the clinical significance of TSAb levels in patients with HT is unclear. Moreover, there are few reports on patients who have transitioned from HT to GD as well as on the characteristics of patients with GD who are prone to this alteration in pathology. The factors for the difference in the disease direction also remains unknown. We encountered cases of patients who underwent a conversion from HT to GD within 2 months and observed changes in laboratory findings before and after conversion. Furthermore, to elucidate the clinical features of these patients, we considered all reported cases of HT that preceded GD and the factors involved in this pathological change.

## 2. Literature Review

Relevant studies were identified through literature search using the PubMed and EMBASE databases. We used the keywords “transition from HT to GD or “GD following HT” or “HT converting to GD” or “HT to GD”. To date, 50 cases regarding conversion from HT to GD have been reported [4,5,6,7,8,9,10,11,12,13,14,15,16,17,18,19,20,21], including this case (Table 1). The male-to-female ratio was 1:9, median age was 44 years (range, 23–82 years), and the most common age group was 36–55 years. Of the 50 reports, 33 (66%) were from Europe and the United States and 17 (34%) were from Asia and Africa. No information regarding racial susceptibility was available.

The median duration of HT before the shift to GD was 7 years (range, 0.1–27 years). A total of 2 of the 50 patients (4%) had a family history of thyroid diseases and 3 of the 38 patients (7.8%) had thyroid ophthalmopathy at the time of HT. This was higher than the 4.3% incidence of thyroid ophthalmopathy in patients with HT without conversion to GD [22].

TSAb levels were analyzed in 11 cases at both HT and GD diagnoses, of which, 8 (73%) were negative at HT and positive during GD. The remaining three patients (27%) were TSAb or thyroid stimulating immunoglobulin (TSI)-positive, which is commonly seen in GD, including the present case. One patient developed GD after pregnancy and was TSAb-positive at the time of HT. One patient had mild thyroid ophthalmopathy at HT, developed GD after human immunodeficiency virus treatment, and was TSI-positive from HT. Additionally, all 50 patients were positive for TSAb or thyrotropin receptor antibody (TRAb) following the shift to GD. In all 50 patients, levothyroxine supplementation was discontinued for at least 4 weeks from the onset of post-diagnostic hyperthyroidism to rule out iatrogenic hyperthyroidism.

All 50 patients received T4 replacement therapy as a treatment for HT. A total of 42 (87.5%) of the 48 patients, excluding 2 unspecified patients, were treated with antithyroid drugs for GD. Twelve patients (25%) subsequently underwent radioactive iodine therapy, and one (2.3%) underwent a thyroidectomy. A total of 2 of the 42 patients (4.7%) treated with antithyroid drugs discontinued drug treatment due to neutropenia. A total of 6 of the 48 patients were not treated with antithyroid drugs, including 5 (10.4%) who were only observed; of these, 4 had euthyroidism and 1 had hypothyroidism, while the remaining patient underwent a total thyroidectomy. After treatment or follow-up for GD, 12 of the 48 (25%) patients showed recovery of thyroid function, whereas 36 (75%) showed hypothyroidism.

## 3. Case Report

A 50-year-old woman presented at our hospital with complaints of ongoing fatigue for 2 months, a 7 kg weight loss, and trunk muscle cramps. The patient had no notable medical or family history of thyroid disease. Physical examination showed myxedema of the face and legs, and biochemical examination revealed elevated blood TSH and low blood triiodothyronine (T3) and thyroxine (T4) levels (Table 2).

**Figure 1:**
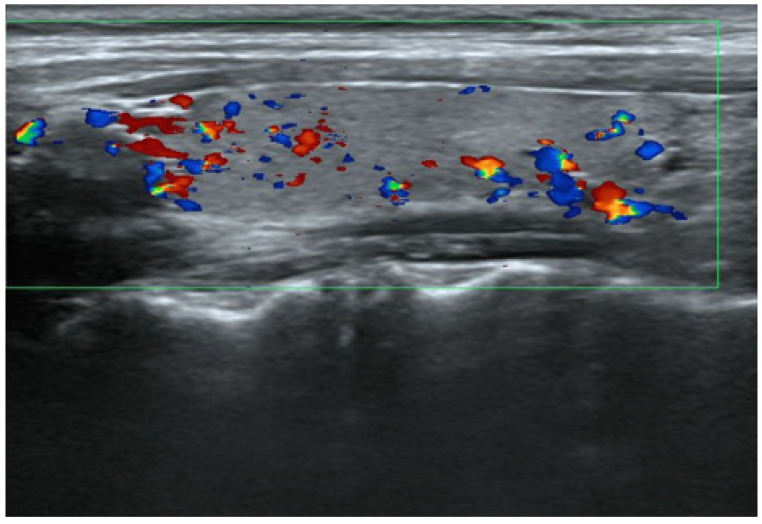
Thyroid US at the onset of GD. The right lobe in the sagittal view shows normal echogenicity and mild hypervascularity. The size of the right lobe was 1.18 × 1.31 × 4.32 cm (volume 3.5 cm^3^) and within normal range.

**Figure 2:**
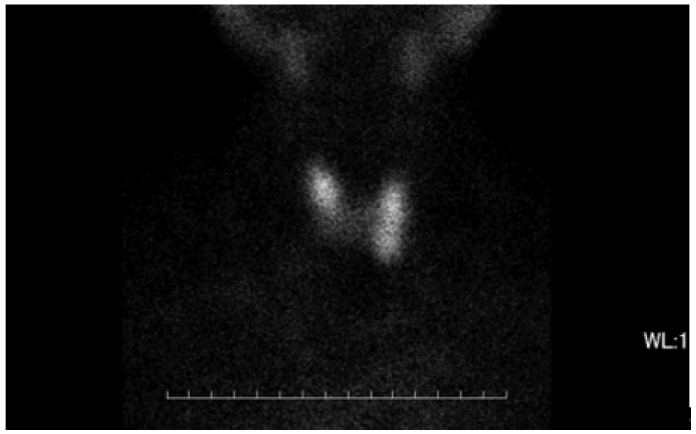
The image of thyroid gland in 99mTc scan. No remarkable accumulation of 99mTc was observed focally or in the whole thyroid gland. The uptake ratio was 0.76% at 30 min, which was within normal range.

The thyroid autoantibody profile was positive for TPOAb, TgAb, and TSAb and negative for TRAb, and the patient was diagnosed with primary hypothyroidism due to HT. Initiation of 50 μg levothyroxine supplementation resulted in a rapid resolution of symptoms and thyroid function normalized. Nine weeks after treatment, the patient experienced palpitation and hyperhidrosis. Consequently, she was diagnosed with clinical hyperthyroidism based on low TSH levels and high T3 and T4 levels. Subsequent discontinuation of levothyroxine for 5 weeks did not affect thyroid function, and serum thyroglobulin (Tg) levels were within the reference range, ruling out iatrogenic hyperthyroidism and destructive thyroiditis. TSAb, TPOAb, and TgAb were consistently positive at the time of HT diagnosis. Thyroid ultrasonography (US) showed mild hypervascularity in the thyroid (Figure 1), as seen in GD, and a technetium-99m pertechnetate (99mTc) scan displayed normal uptake, different from nodular accumulation as a functional tumor or decreased accumulation as destructive thyroiditis (Figure 2).

Based on the above, the patient was diagnosed with GD, and treatment with methimazole (MMI) 5 mg began on day 129 of the HT diagnosis. Thyroid function normalized on day 150; however, mild neutropenia and elevated TSH levels developed on day 171, and the dose of MMI was reduced to 2.5 mg. On day 198, TSH levels decreased back within the reference range, and MMI treatment was withdrawn. Thus, neutropenia was treated. TSAb levels decreased gradually during the treatment period but remained positive 7 months after HT diagnosis.

## 4. Discussion

In this report, we reviewed all previous studies and described a case of a patient with a transition from HT to GD in 65 days. Initially, the patient was diagnosed with HT based on clinical findings and biochemical parameters including elevated serum TSH levels and high titers of TPOAb and TgAb. Subsequently, she developed clinical hyperthyroidism, with increased fT3 and suppressed TSH. Hyperthyroidism persisted 2 months after thyroxine was discontinued, and TSAb remained positive, leading to the diagnosis of GD. Iatrogenic hyperthyroidism due to excessive thyroxine replacement was excluded, since the period without treatment was long enough to evaluate the clinical state. Destructive thyroiditis was also excluded, as the increased fT3 and fT4 persisted for 2 months accompanied by Tg within normal range. However, the increased uptake of 99mTc into the thyroid, typically seen in many cases of GD, was not observed. GD-induced hyperthyroidism was evident and required antithyroid therapy. MMI immediately improved her thyroid function and thus was withdrawn due to neutropenia.

This study also reviewed previous reports of HT preceding GD to identify the clinical features. Patients with HT preceding GD received thyroid hormone replacement therapy for hypothyroidism prior to the onset of GD. In addition, HT before transition to GD had a higher incidence of thyroid ophthalmopathy than HT without GD conversion. TSAb was also positive in HT preceding GD, consistent with common GD. It is likely that TSAb appeared in the course of HT, leading to the development of GD. Nearly 10% of cases showed spontaneous remission of GD. It was also notable that neutropenia, a side effect of antithyroid drugs, was higher in cases of HT-preceded GD than in cases of general GD.

GD is characterized by hyperthyroidism resulting from an autoimmune process and the presence of TRAb. TRAb have a high affinity for the TSH receptor with two different effects: stimulation and inhibition. In GD, the stimulatory effects on TSH receptors, typified by TSAb, lead to unregulated hypersecretion of the thyroid hormone, comparable to stimulatory TRAb. In contrast, the inhibitory effect of TSH on receptor binding, detected as TSH-stimulation blocking antibodies (TSBAb) and thyrotropin binding inhibiting immunoglobulins (TBII), causes hypothyroidism in patients with autoimmune thyroiditis with a different pathological background than that of HT. Thus, the conversion between hypothyroidism and hyperthyroidism in TRAb-positive GD reflects a push–pull effect between inhibitory and stimulatory antibodies. The clinical presentation is determined by the predominant antibody and varies with time among patients [13,23] in the entire observation period in this case; therefore, this mechanism cannot be applied to all cases of HT conversion to GD. Multiple factors are reportedly involved in the conversion of these two types of autoimmune thyroiditis. Whether the disease is directed towards GD or HT is influenced by the balance of Th1/Th2 cytokines within the thyroid tissue. Th1 is the predominant lymphocyte in HT [24]. Additionally, GD as a T2-driven disease has become controversial, with some reports disproving this hypothesis, reinforcing a priority role of Th1 [25,26]. High levels of Th1 chemokine receptor are present in patients with GD with an active phase. Recent studies have also showed that MMI progressively induced transition from Th1 to Th2 predominance [27]. Therefore, the autoimmune response in thyroid-specific autoimmune disorders includes components of both Th1 and Th2 types. Cytokines and/or chemokines were not examined in this case. However, previous studies suggest that the dominant lymphocyte in the thyroid tissue of the present case was Th1, and the pathology shifted from HT to GD. In addition, age-related immunological changes are a driving factor that causes the conversion, and people aged 80 years and above are more likely to experience the conversion [4]. Immunomodulators such as alemtuzumab are also known to cause thyrotoxicosis [28]. These factors, however, are not necessarily applicable in previous reports, including the present case.

Furthermore, TSAb are detected in 90% of GD cases and 10% of HT cases [29]; however, the number of cases of HT converting to GD reported so far is small. Additionally, the complication rate of thyroid ophthalmopathy in HT shifting to GD later is 7.8%, higher than the 4.3% observed in HT without conversion to GD [30]. It is considered that the patients with HT preceding GD had positive TSAb levels in the period of HT diagnosis, since TSAb is associated with the development of thyroid ophthalmopathy. Therefore, the prevalence of TSAb-positive patients is possibly higher than previously reported. The paucity of reported cases of transition from HT to GD suggests that TSAb is not the sole determinant of disease transition.

Whether TSAb-positive patients with HT have the ability to secrete hormones from thyroid follicular cells is an important factor to consider in the conversion from HT to GD. Exposure of thyroid follicular cells to TPOAb and TgAb induces intracellular lymphocytic infiltration and irreversible hypofunction owing to fibrosis and atrophy [22,31]. Our patient presented with severe hypofunction, which later converted to hyperfunction due to GD. Therefore, the thyroid follicular cells, in this case, regained the ability to secrete thyroid hormone in the stage of GD after HT. The thyroid US, in this case, displayed an average size without atrophy. This confirms that the thyroid had not yet reached the final stage of HT with irreversible changes present.

Among the 50 patients in the literature, TSAb during the HT period was unmeasured in 39, negative in 8, and positive in 3, including this case. TSAb levels in eight cases turned positive in the GD period. Three patients, including the present case, remained positive during both HT and GD. A previous report stated that TSAb was positive at least 2 months prior to the onset of GD; however, it is unclear when TSAb in other cases was measured during HT. In HT preceding GD, the median period of conversion from HT to GD was 7 years; only a few cases had a conversion period as short as 2 months, as in our case. It is likely that TSAb production, thus, starts before the appearance of clinical hyperthyroidism caused by GD, since TSAb is detected in the majority of general GD cases. Consequently, long-term or regular monitoring of TSAb in HT may help to identify the patients with a risk of transition to GD.

Additionally, TPOAb and TgAb are detected in 95% and 60–80% of HT patients, respectively [32]. These autoantibodies are major factors in the pathogenesis of HT. Conversely, 50–70% of GD patients show positive TPOAb and TgAb, and these increase the possibility of conversion of GD to HT after treatment with antithyroid drugs. However, the clinical significance of these antibodies in GD remains unclear [33].

It may seem paradoxical that the thyroid gland in HT responds to stimulation by TSAb and produces excessive hormone, despite the lack of response to stimulation by excess TSH. This mechanism can be explained by the higher affinity of TSAb as TSH receptor agonists than TSH [34]. Therefore, hypersecretion occurs under intensive stimulation by TSAb, even if the thyroid gland has a poor ability to produce hormones. 

Another factor related to thyroid responsiveness in HT conversion to GD is the effect of T4 replacement during recovery of thyroid function. All cases in the literature received T4 replacement therapy. HT patients receiving T4 replacement may develop GD under positive TSAb levels [35]. Additionally, T4 replacement reportedly increases both TSAb positivity and antibody titers in patients with hypothyroidism due to blocking of TSBAb [24,30]. These studies indicate that replacement therapy for hypothyroidism may influence the emergence of TSAb, resulting in the development of GD. However, it should be noted that not all patients who developed GD following HT showed TSAb positivity.

The male-to-female ratios in patients with general GD and HT are 1:10 and 1:18, respectively. GD usually occurs at a young age, and has a peak of 30–60 years, whereas HT occurs in middle-aged individuals around 40–60 years. In the literature, most patients were female, with a male-to-female ratio of 1:9, and the age of GD onset was 36–55. In terms of sex differences, the incidence of HT preceding GD was close to that of general GD, and the age of onset was similar to that of general HT and GD. As mentioned above, patients converting from HT to GD have not been reported as frequently as those converting from GD to HT and have not been defined. One possible explanation for this discrepancy is the difference in the clinical presentation between GD and HT. Hyperthyroidism in GD interrupts daily activity due to the overt symptoms, such as palpitations, hyperhidrosis, weight loss, and finger tremors. On the other hand, symptoms associated with HT are generally vague and include non-specific findings such as malaise, coldness, and facial edema. Therefore, it is possible that HT prior to GD was not evaluated and GD in the same individual was solely diagnosed. According to this hypothesis, the number of patients who develop GD following HT is higher than reported. The male-to-female ratio, 1:9, in HT preceding GD supports this theory, since it is closer to the ratio in general GD than in general HT. 

Most cases of HT preceding GD were treated with MMI, in which the adverse effect of neutropenia was reported. Dose-dependent neutropenia occurs within 3–4 months after MMI initiation [36]. Moreover, neutropenia in HT preceding GD was observed in two cases, in a report by Clifford and Wakil [10] and the present case. Neutropenia occurred on day 26 of treatment with 25 mg carbimazole and on day 41 of 5 mg MMI, respectively. The rate of neutropenia as an adverse effect of antithyroid drugs in GD shifted from HT was 4.7%, higher than in general GD (0.1–0.5%) [37]. In addition, five cases (10.4%) of HT preceding GD spontaneously improved without antithyroid drugs or radioactive iodine, whereas GD generally requires long-term treatment. This result suggests that patients of HT preceding GD may have more opportunities to achieve remission than in general GD. Thus, some cases with GD following HT are potentially transient and simply need symptomatic therapy and observation. 

This study could not clarify the determinants of pathology in cases of autoimmune thyroiditis that converted from HT to GD. One of the limitations of this study is that omics analysis was not performed and the molecular basis of the conversion from HT to GD could not be clarified, as the use of samples including blood specimens was beyond the scope of this study.

Based on the above, we provide clinically useful observations of HT-to-GD autoimmune thyroiditis. In patients with HT, the possibility of conversion to GD should be considered if TSAb is positive after the initiation of replacement therapy. Hyperthyroidism is often transient, and some patients do not require thyroid therapy. Additionally, the incidence of neutropenia, caused by antithyroid drugs, is similar in HT-preceded GD and general GD. Therefore, these factors should be considered when selecting treatment for HT preceding GD. Moreover, chronological observation of TSAb levels may also be useful in predicting conversion to GD.

## 5. Conclusions

HT preceding GD can be a subtype of autoimmune thyroiditis, as not all patients with HT convert to GD. According to our speculation, HT preceding GD occurs more frequently in a clinical background than has been reported. The clinical characteristics do not necessarily match those reported in HT or GD, suggesting the need to be especially careful when determining treatment strategies. Close observation and monitoring of TSAb in HT patients will help to increase the reported cases of conversion from HT to GD and clarify the clinical presentation.

## Figures and Tables

**Table 1:** Conversion to Graves’ disease from Hashimoto’s thyroiditis: reported cases.

Author	Number of Patients	Age (Years)	Sex	Duration of HT before GD Onset	TSAb		TRAb	Treatment of GD	Outcome
					HT State	GD State	GD State		
Kamath et al. [4]	1	80	F	20 years	NA	NA	Pos	Antithyroid drug, radioiodine	Hypothyroidism
Kraiem et al. [5]	1	55	F	3 years	NA	Pos	NA	Antithyroid drug, radioiodine	Hypothyroidism
Ahmad et al. [6]	1	60	F	27 years	NA	NA	Pos	Antithyroid drug	Euthyroidism
Bishay and Chen [7]	1	60	M	1 year	NA	NA	Pos	Antithyroid drug	Euthyroidism
Cho et al. [8]	1	40	M	1 year	NA	Pos	ND	NA	NA
Chung et al. [9]	1	60	M	6 years	NA	NA	Pos	Antithyroid drug	Euthyroidism
Clifford and Wakil [10]	1	51	M	6 years	NA	NA	Pos	Antithyroid drug, total thyroidectomy	Hypothyroidism
Ekpebegh et al. [11]	1	54	F	5 years	NA	NA	Pos	Antithyroid drug	Euthyroidism
Fan et al. [12]	1	52	F	20 years	NA	Pos	ND	Antithyroid drug, radioiodine	Hypothyroidism
Furqan et al. [13]	3	36	F	6 years	NA	NA *	NA *	Antithyroid drug	Hypothyroidism
		46	F	2 years	NA	NA *	NA *	Antithyroid drug, radioiodine	Hypothyroidism
		43	F	1 month	NA	NA *	NA *	Antithyroid drug	Euthyroidism
Gonzalez-Aguilera et al. [14]	24	Mean 39	22 F 2 M	Mean 3 years	NA	NA	Pos	Antithyroid drug, radioiodine ^†^	Hypothyroidism
Kasagi et al. [15]	1	23	F	ND	NA	Pos	NA	Antithyroid drug	Euthyroidism
Takasu et al. [16]	7	23	F	1 year	Neg	Pos	NA	No treatment	Euthyroidism
		53	F	1 year	Neg	Pos	NA	No treatment	Euthyroidism
		28	F	2 years	Neg	Pos	NA	Antithyroid drug	Euthyroidism
		24	F	2 years	Neg	Pos	NA	No treatment	Hypothyroidism
		38	F	1 year	Neg	Pos	NA	No treatment	Euthyroidism
		42	F	2 years	Neg	Pos	NA	Antithyroid drug	Euthyroidism
		38	F	ND	Neg	Pos	NA	Antithyroid drug	Euthyroidism
Pak et al. [17]	1	41	F	ND	NA	Pos	NA	Total thyroidectomy	Hypothyroidism
Watari and Jassil [18]	1	56	F	16 years	Pos ^a^	Pos	NA	Symptomatic treatment	Euthyroidism
Takeda et al. [19]	1	48	F	2 years	Neg	Pos	NA	NA	NA
Kempegowda and Nayak [20]	1	62	F	25 years	NA	NA	Pos	Antithyroid drug, radioiodine	Hypothyroidism
Lu et al. [21]	1	34	F	4 years	Pos	Pos	Pos	Antithyroid drug	Hypothyroidism
Current case	1	50	F	2 months	Pos	Pos	Neg	Antithyroid drug	Euthyroidism

HT, Hashimoto’s thyroiditis; GD, Graves’ disease; F, female; M, male; TRAb, thyrotropin receptor antibody; TSAb, thyroid-stimulating antibody; Neg, negative; Pos, positive; NA, not available; ND, not determined. ^a^ Positive thyroid stimulating immunoglobulin. * Positive TRAb and/or TSAb. ^†^ Antithyroid medication was given to seven individuals. If thyroid function did not improve, radioiodine was the only curative choice; 17 patients received only antithyroid medication.

**Table 2:** Biochemical changes of thyroid function.

	Day 1	Day 23	Day 65	Day 86	Day 94	Day 129	Day 150	Day 171	Day 198	Day 217
**TSH (0.45–3.72 μIU/mL)**	115.26	14.22	0.449	0.099	0.139	0.046	2.832	6.567	2.729	1.127
**fT3 (2.1–3.1 pg/mL)**	1.65	2.39	3.22	4.00	3.62	4.27	2.57	2.45	2.68	2.84
**fT4 (0.75–1.42 ng/mL)**	0.25	0.93	1.55	1.99	1.35	1.91	0.91	0.96	1.26	1.04
**Tg (<33.7 ng/mL)**	5.76	2.34	2.34	3.63	4.34	5.02	ND	6.81	ND	5.25
**TRAb (<2 IU/L)**	1.8	1.9	1.3	1.4	1.1	1.9	ND	2.0	ND	1.6
**TSAb (<120%)**	443	ND	163	142	181	181	ND	184	ND	155
**TPOAb (<16 IU/mL)**	183.4	134.5	73.3	73.4	60.3	36.1	ND	30.8	ND	22.9
**TgAb (<28 IU/mL)**	309.3	253.9	239.1	256.4	247.3	223.3	ND	190.2	ND	154.4
**Treatment**	LT4		none			MMI 5 mg		MMI 2.5 mg	none	

TSH, thyroid-stimulating hormone; fT3, free triiodothyronine; fT4, free thyroxine; Tg, thyroglobulin; TRAb, thyrotropin receptor antibody; TSAb, thyroid-stimulating antibody; TPOAb, thyroid peroxidase antibody; TgAb, thyroglobulin antibody; LT4, levothyroxine; MMI, methimazole; ND, not determined.

## Data Availability

Not applicable.
